# Polypi of the Female Urethra

**Published:** 1846-08

**Authors:** 


2. *Polypi of the Female Urethra.*—In our preceding number (p. 484,) will be found some interesting remarks by Dr. Lever, on the vascular growths which occur in the female urethra, an affection to which sufficient attention has not been directed. With a view of furnishing a more complete history of the disease, we shall now present a summary of an interesting thesis (*Théses de Strasburg*, No. 129), by Dr. M. H. Bavoux, as given in the *Archives Generales*, for September last.

In the female urethra both a mucous and vascular structure exists, and this peculiarity gives to the polypi which occur in it, a peculiar character. They possess both a mucous and vascular structure, and these two characteristic elements are never observed increasing independently of each other. On the contrary, these small bodies are constantly seen to originate from hypertrophy of the mucous membrane, into which numerous vessels from the subjacent erectile tissue are prolonged, so that of all the species of polypi described by authors, those which, in a descriptive point of view, approach nearest to these tumors of the urethra, are, without doubt, the fungoid species; with this distinction, however, that these polypi rarely, and as it were exceptionally, degenerate.

Polypi of the urethra very rarely occur before the age of puberty, and appear to have for their cause, a too great stimulus of the genital organs. Thus the affection is more frequently met with in prostitutes than in other females. Schutzemberger has seen them occcur after blennorrhagia; but, of course, frequent coitus or masturbation may act in the same manner.

The polypi sometimes project beyond the orifice of the urethra, and lie between the large labia; they are sometimes retained within the interior of the canal; and hence the division into *external* and *internal* polypi.

*External polypi* are of much more frequent occurrence than the latter, and generally originate from the posterior wall of the canal, near the meatus urinarius, a circumstance which did not escape the observation of Boyer. At other times, however, they originate higher up, and thus remain concealed for a longer or shorter period, till by their increase or size, or the elongation of their pedicle, they at length protrude. Their size is seldom considerable; it varies from that of a currant to that of a large cherry, and rarely exceeds that of the latter. The pedicle is in general large as compared with that of the polypus, and decreases in size as the latter enlarges. Their shape at the commencement is very generally that of a cone; at a later period, from the increase of growth being irregular at various points, they assume a lobulated appearance. Their surface most generally is of a bright red colour; at other times it is somewhat pale, and at others of a deep red: sometimes they are entirely covered with a thin smooth epithelium; at others, this is wanting, and then they exhibit a villous fungoid aspect, similar to that of a wound in a state of suppuration. In this case, the tumour bleeds more easily when touched, is more painful, and smarts from contact with the urine. In general, polypi of the urethra causes no pain; in some cases, however, a sensation of burning, or even of extreme pain, is produced after walking, coitus, or the passage of the urine; the pain may extend to the fundus of the bladder, rectum, or uterus, so as to lead to a suspicion of disease in the latter. They are sometimes the cause of hematuria, and very often of slight hemorrhage after coitus. In some cases there is over-excitement of the genital organs; but there is rarely any difficulty in passing urine, and still more rarely incontinence of urine, even in those cases where the urethra is so much dilated as to permit the entrance of the finger. At first, their growth is pretty rapid, but after attaining the size of a bean or cherry, it becomes slower, or they remain altogether stationary. In one case in which the author saw the tumor developed, as it were, under his eyes, its commencement was marked by the rise of groups of vascular granulations on the lower wall of the meatus; these granulations become united at their base; the interstices which existed between them were filled up, and thus a polypus of the size of a pea was formed, with a tolerably large pedicle; it was excised by means of a pair of scissors. In general, polypi of the urethra are not a serious affection and may continue for an indefinite period. Spalderer, according to M. Gerdy, saw one evacuated with the urine. M. Tanchou, on the other hand, regards it as a disease very difficult to be rooted out in adult females; but it is probable that the cases witnessed by M. T. were not cases of true polypus of the urethra.

*Internal polypi* seldom occasion any well-marked symptom which may lead to the suspicion of their existence; after attaining a certain size they make their appearance externally and then come under the description of those we have just given.

The author has traced, with great care, the differential diagnosis between polypus of the urethra, and some other affections for which it may be mistaken. The absence of accurate diagnosis is of little importance, as far as regards distinguishing it from hernia and hypertrophy, either of the mucous membrane or its folds; but the case is very different as regards introversion of the fundus of the bladder, or venereal vegetations. Introversion of the fundus of the bladder is characterized by the presence of a soft reducible tumour, of the size of a nut, and of a bright red colour; it is accompanied with severe pain and dysuria, which disappear after the introduction of a sound into the urethra. Polypi, on the other hand, are soft, indolent, irreducible tumours, which occasion no inconvenience in the excretion of the urine, and offer no obstacle to the introduction of the catheter into the urethra. An error in diagnosis between these two affections is easily committed, and if not avoided would lead to danger; more especially if excision of the tumour were attempted; but an error scarcely less unfortunate is that of mistaking polypi for venereal vegetations, not only because it throws suspicion on an otherwise innocent person, but also as subjecting her to a course of general treatment not altogether void of danger. An attentive examination of the tumour ought to remove all doubt. How, indeed, can a solitary, pediculated tumour, of a redder colour than the membrane, but having its consistence, bleeding easily, with a regular or lobulated surface, of a smooth or shreddy character, but always soft, be confounded with those small, hard, unequal projections of the size of a pin's head or a hemp seed, or with red granular excresences, or finally, with those small flattened prolongations of the mucous membrane of the vulva, with irregular edges?

*Treatment.*—The author has never seen any good result from topical applications, (acetate of lead, for instance,) or repeated cauterizations. Pressure by means of conical bougies introduced into the urethra, as advised by Madame Boivin, has likewise appeared to him of little benefit. Removal of the tumor, either by scissors or ligature, is the only treatment he has found to be efficacious. In a case still under treatment, the tumour separated four days after the application of the ligature. Excision is more expeditious and less painful. It may be performed with a pair of curved scissors the tumour having been previously seized with a pair of forceps, or a thread passed through its pedicle, so as to drag it outside the canal. In cases of internal polypus, the canal must be previously dilated, or an incision made through its walls, as performed by Varner, ere its excision can be attempted. As a matter of prudence the point of attachment of the pedicle ought to be cauterized, in order to prevent any tendency to reproduction.—*Amer. Jour. of Med. Sciences.*

